# Reversion and T Cell Escape Mutations Compensate the Fitness Loss of a CD8^+^ T Cell Escape Mutant in Their Cognate Transmitted/Founder Virus

**DOI:** 10.1371/journal.pone.0102734

**Published:** 2014-07-16

**Authors:** Hongshuo Song, Bhavna Hora, Tanmoy Bhattacharya, Nilu Goonetilleke, Michael K. P. Liu, Kevin Wiehe, Hui Li, Shilpa S. Iyer, Andrew J. McMichael, Alan S. Perelson, Feng Gao

**Affiliations:** 1 Duke Human Vaccine Institute, Duke University Medical Center, Durham, North Carolina, United States of America; 2 Theoretical Division, Los Alamos National laboratory, Los Alamos, New Mexico, United States of America; 3 Santa Fe Institute, Santa Fe, New Mexico, United States of America; 4 Weatherall Institute of molecular Medicine, University of Oxford, Oxford, England, United Kingdom; 5 Departments of Medicine and Microbiology, University of Pennsylvania, Philadelphia, Pennsylvania, United States of America; Institut Pasteur, France

## Abstract

Immune escape mutations that revert back to the consensus sequence frequently occur in newly HIV-1-infected individuals and have been thought to render the viruses more fit. However, their impact on viral fitness and their interaction with other immune escape mutations have not been evaluated in the background of their cognate transmitted/founder (T/F) viral genomes. To precisely determine the role of reversion mutations, we introduced reversion mutations alone or together with CD8^+^ T cell escape mutations in their unmodified cognate T/F viral genome and determined their impact on viral fitness in primary CD4^+^ T cells. Two reversion mutations, V247I and I64T, were identified in Gag and Tat, respectively, but neither had measurable effect on the fitness of their cognate T/F virus. The V247I and G248A mutations that were detected before and concurrently with the potent T cell escape mutation T242N, respectively, were selected by early T cell responses. The V247I or the G248A mutation alone partially restored the fitness loss caused by the T242N mutation. Together they could fully restore the fitness of the T242N mutant to the T/F level. These results demonstrate that the fitness loss caused by a T cell escape mutation could be compensated by preexisting or concurrent reversion and other T cell escape mutations. Our findings indicate that the overall viral fitness is modulated by the complex interplay among T cell escape, compensatory and reversion mutations to maintain the balance between immune escape and viral replication capacity.

## Introduction

The mutations selected in previous donors often revert back to the consensus sequence after the viruses are transmitted into a new host [1]–[9]. The well-studied cases are the reversions of CD8^+^ T cell escape mutations after the escape mutant viruses are transmitted into HLA-mismatched recipients [2], [5]–[7]. Reversions are also detected at positions for which CD8^+^ T cell immune selection cannot be defined [2], [4], [8], [9]. It has been assumed that reversion mutations render the viruses more fit [1], [5], [6]. However, the fitness impact of reversion mutations has not been evaluated in their cognate or heterologous viral genomes. It generally takes more than a year for the reversion mutations to fully replace the transmitted virus population in the new hosts [5], [10]–[12] and some reversions only transiently occur in the viral population [13]. These observations suggest that reversion may not generally render their cognate transmitted/founder (T/F) viruses significantly more fit. However, their impact on viral fitness remains to be elucidated.

Previous studies showed that some CD8^+^ T cell escape mutations could led to fitness loss [10], [14]–[17], while some of others were without any measurable fitness cost in the *in vitro* fitness assays, especially for the CD8^+^ T cell escape mutations in Env [18], [19]. The fitness-impairing CD8^+^ T cell escape variants may contribute to better viral load control and improved clinical outcome [14], [16], [20], [21], and when transmitted, may provide clinical benefit to the recipients [10], [12], [17], [22]. Therefore, CD8^+^ T cell escape mutations that can significantly reduce viral fitness can serve as ideal targets for vaccines to select in order to reduce viral fitness, which then may slow disease progression and reduce transmission rates. However, a recent study showed that there was no long-term protection against HIV disease progression in HLA-matched individuals infected with CD8^+^ T cell escape variants, and the reversion of CD8^+^ T cell escape mutations was associated with the increase in viremia in HLA-mismatched recipients [12]. Some studies showed that the fitness loss of CD8^+^ T cell escape mutations could be partially or fully recovered by compensatory mutations in and outside the T cell epitopes [15], [23]–[25], and the restored fitness might reduce the clinical benefit conferred by the CD8^+^ T cell escape mutations [15], [26]–[28]. Thus, it is critical to understand how exactly fitness costs of CD8^+^ T cell escape mutations can be restored by compensatory mutations, and identification of the fitness-impairing CD8^+^ T cell escape mutations that cannot be compensated may have important implications for vaccine design.

A better understanding of the impact of immune escape, compensatory mutations and reversions on viral fitness, as well as their interplay in shaping the balance between immune escape and viral survival could be an important step towards a better understanding of disease control and identification of epitopes for vaccine design. In our previous study, we found that a virus carrying CD8^+^ T cell escape mutations together with reversion mutations was as fit as their cognate T/F virus using our newly developed PASS fitness assay [25]. In this study, we further investigated how the reversions and CD8^+^ T cell escape mutations interact with each other and compensate viral fitness loss by studying them individually and in combination in their unmodified cognate T/F viral genome.

## Materials and Methods

### Infectious molecular clones and viral stocks

The infectious molecular clone (IMC) for the CH77 T/F virus was chemically synthesized in a previous study [29]. The mutations were introduced into the CH77 T/F IMC by site-directed mutagenesis (Stratagene Santa Clara, CA). The virus stocks were generated by transfecting the IMCs into 293T cells as previously described [30].

### Viral replication kinetics in CD4^+^ T cells

Peripheral blood mononuclear cells (PBMC) were obtained through leukophereses from healthy donors as previously described [25]. The written consent was obtained from the donors and the study was approved by the Duke University Institutional Review Board. PBMCs were isolated using the Ficoll-Hypaque density gradients and lymphocytes were isolated by elutriation using standard techniques. CD4^+^ T cells were negatively selected from PBMCs or lymphocytes on an autoMACS Pro Separator using the CD4^+^ T cell Isolation Kit II (Miltenyi Biotec, Auburn, CA). Purified CD4^+^ T cells were stimulated for 3 days in RPMI1640 containing 10% fetal bovine serum (FBS), interleukin 2 (IL-2) (32 IU/ml; Advanced Biotechnologies, Columbia, MD), soluble anti-CD3 (0.2 µg/ml; eBioscience, San Diego, CA) and anti-CD28 (0.2 µg/ml; BD Bioscience, San Diego, CA). The stimulated CD4^+^ T cells (1×10^6^) were infected with 1000 TCID_50_ of each virus (m.o.i≈0.001). After an incubation at 37°C for 4 hours, the cells were washed 3 times with RPMI 1640, and cultured in a 24-well plate in 600 µl of RPMI 1640 containing 10% FBS and IL-2 (32 IU/ml). Each virus was cultured in triplicates. Viral replication was monitored daily for 5 days by measuring the p24 concentration in the culture supernatant with the p24 ELISA kit (PerkinElmer, Waltham, MA).

### Competitive PASS fitness assay

Competitive fitness assay was performed as described in our previous study [25]. In brief, after stimulation with soluble anti-CD3 and anti-CD28, 50 µl of cell suspension (1×10^6^) was seeded into each well of a 96-well plate and infected with the virus mixture stock containing the viruses to be compared (5 ng p24 of each virus). After absorption at 37°C for 4 hours, the cells were washed 3 times with RPMI 1640. The infected cells were cultured in a 24-well plate with 600 µl of RPMI 1640 containing 10% FBS and IL-2 (32 IU/ml). In the single-passage assay, the culture supernatant was harvested and replaced with fresh medium daily. The kinetics of virus replication was monitored by measuring the p24 concentration in the supernatant. In the multiple-passage assay, the first round of infection was carried out as in the single-passage infection described above. The supernatant was harvested at day 3 or day 4 at the peak of the p24 production, and 200 µl of the supernatant was passaged onto fresh CD4^+^ T cells (about 10 ng p24 per 10^6^ cells). The viral replication at each passage was monitored by measuring the p24 concentration. All infections were performed in triplicate.

The cell culture supernatants were treated with RNase-free DNase to eliminate the residual plasmid DNA used for transfection. Viral RNA was then extracted from 100 to 200 µl of the culture supernatant using the PureLink Viral RNA/DNA Mini Kit (Invitrogen, Carlsbad, CA). RNA was eluted into 20 µl of RNase-free water. The viral RNA (17 µl) was used for cDNA synthesis using SuperScript III reverse transcriptase (Invitrogen, Carlsbad, CA) with the primer A4-lower: 5′-GAGTAAATTAGCCCTTCCAGTCC-3′ (nt 9082–9104 in HXB2) for detection of mutations in the *tat* and *env* genes and the primer A1-lower: 5′-CACAGGAACAAGCAGCCAGGTC-3 (nt 1152–1173) for detection of the mutations in the TW10 epitope in the *gag* gene. The cDNA was stored at −20°C for later use.

The parallel allele-specific sequencing (PASS) assay was performed as described previously [25], [31]. The tat/env fragment was amplified by using the PCR primers R-lower (5′ Acry-GGAAGCACCCAGGAAGTCAGC-3′; nt 5862–5882) and R-upper (5′-GTATCCTCTGATGGGAGGGGCATA-3′; nt 7527–7550), and the amplicons were annealed with the sequencing primer Rev7 (5′-ATGCTACTTACTGCTTTGGTAGAGGCGCTTGATTA-3′; nt 6022–6056) to detect the I64T mutation or the sequencing primer Rev13 (5′-CCTCCTGAGGAATGGTTAAAGACTAT-3′; nt 7299–7324) to detect the R355K mutation. The gag amplicon was amplified by the primers A1-lower (5′ Acry-AGGGGTCGTTGCCAAAGAGTGA-3′; nt 2260–2281) and A1-upper (5′-CACAGGAACAAGCAGCCAGGTC-3′) and the amplicons were annealed with the sequencing primer C1548A (5′-AAGGGGAAGTGATATAGCAGGATCTACTAGTA-3′; nt 1482–1513) to detect the T242N mutation or G1562A (5′-TATAGCAGGATCTACTAGTACCCTTCAGGAACAA-3′; nt 1494–1527) to detect the V247I mutation, or G1566C (5′-CAGGATCTACTAGTACCCTTCAGGAACAAGTAG-3′; nt-1499–1531) to detect the G248A mutation. After PCR amplification, the single base extension (SBE) reaction, gel imaging and data analysis were performed as previously described [25].

### Determination of relative fitness

Relative fitness was determined using a previously developed mathematical model [25] that relied on p24 data to provide a viral population growth rate and PASS assay data to determine the frequencies of the competing variants as a function of time. This model does not assume an exponential growth rate that is constant in time, instead it assumes that the growth rates of the viral variants being compared are proportional to each other at all times, with the proportionality constant determined by the fitness difference. Specifically, if *N_i_ (t)* represents the concentration of the *i* ^th^ virus at time *t,* the model defines 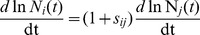, where the relative fitness, *s_ij_*, is assumed independent of time. Note that when *s_ij_* >0, virus *i* grows faster than virus *j*, whereas when *s_ij_* = 0 both viruses grow at the same rate. Each of the replicate experiments was analyzed separately. The PASS assay provided a sample of the viral variants with binomial sampling errors; the concentration of each variant was determined by multiplying the proportion determined from this assay by the total viral concentration inferred from the p24 measurements. Relative fitness was then estimated by fitting the viral concentrations at different times using a maximum likelihood procedure. The estimates from the three replicates were combined using a weighted mean and significance assessed using a t-test. All the statistical comparisons were performed using the statistical package R [32], and the nonlinear fits used the R package subplex [33] and ucminf [34]. Further details about the model equation being fit can be found in [25].

### Structural analysis of the TW10 mutations

Homology models for the p24 monomer of T/F along with T242 and NIA mutants were generated with the Rosetta protein modeling package [35] by threading their respective sequences onto the crystal structure of the full-length 221-residue capsid protein [36] which includes both the N-terminal domain (NTD) and C-terminal domain (CTD).

### Ex vivo IFN-γ ELISpot assay

The T cell response to WT and mutant TW10 peptides (Gag_240–249_) at day 102 and 592 (post Fiebig stage I/II) in CH77 were determined using an *ex vivo* IFN-γ ELISpot assay as we have previously described [9]. ELISpot data are expressed as the mean spot forming units (SFU) per million PBMC (SFU/10^6^ PBMC)±SEM. Positive T cell responses were defined as: ≥30 SFU/million and >4 times above background. All assays were performed in triplicate with a peptide concentration of 2 µg/ml.

## Results

### The V247I or G248A mutation caused partial escape from the early T cell responses

**Figure 1:**
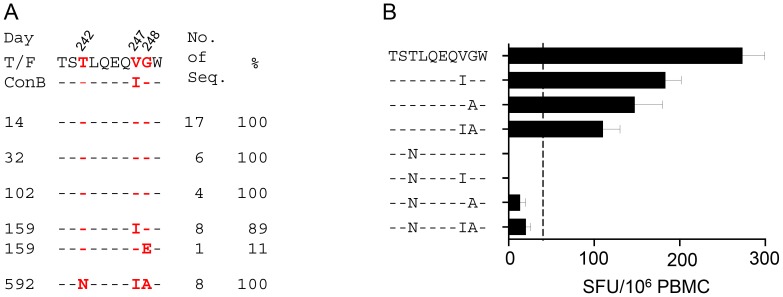
Determination of T cell responses by ELISpot. (A) Frequencies of the mutations in the TW10 epitope at different time points (days post Fiebig stage I/II). The viral sequences obtained by SGA were compared to the T/F virus and the subtype B consensus sequence (ConB). Amino acid substitutions at positions 242, 247 and 248 are highlighted in red. (B) T cell response to the wild type and mutant TW10 peptides at day 102 in subject CH77 were determined using an *ex vivo* IFN-γ ELISpot assay. Peptides containing T242N, V247I or G248A mutation alone as well as in various combinations were analyzed. ELISpot data are expressed as the mean spot forming units (SFU) per million PBMC (SFU/10^6 ^PBMC)±SEM. Positive T cell responses were defined as: ≥30 SFU/million and >4 times above background (indicated by the dotted line). All assays were performed in triplicate.

Three mutations (T242N, V247I and G248A) were detected in all viral genomes in the TW10 epitope at day 592 post Fiebig stage I/II in subject CH77 (Fig. 1A) [9]. Both T242N and G248A mutations were previously identified CD8^+^ T cell escape mutations [5], [9], [37], [38]. The V247I mutation was a reversion mutation towards the subtype B consensus sequence and was recognized equally well by the autologous T cells at day 592 [25]. To determine whether these mutations, individually or in combination, were associated with early T cell responses in CH77, the ELISpot assay was performed with eight 9-mer peptides containing either the wild type (wt) TW10 sequence or various mutations using CH77 PBMC from day 102 (Fig.1B). The peptides containing the V247I mutation, the G248A mutation, or both mutations (V247I/G248A) were partially recognized compared to the wt TW10 peptide. Peptides containing the T242N mutation led to a complete escape from the T cell response (Fig. 1B). These results were in agreement with previous studies, which reported that the G248A mutation alone caused partial loss of the T cell recognition, while the T242N mutation led to a complete escape from the T cell responses targeting the TW10 epitope [5], [9], [25], [37], [38]. Thus, both V247I and G248A were early weak CD8^+^ T cell escape mutations, while the T242N was a potent CD8^+^ T cell escape mutation in CH77.

### The reversion/escape mutation V247I or the CD8^+^ T cell escape mutation G248A partially restored the fitness loss caused by the T242N mutation

**Figure 2:**
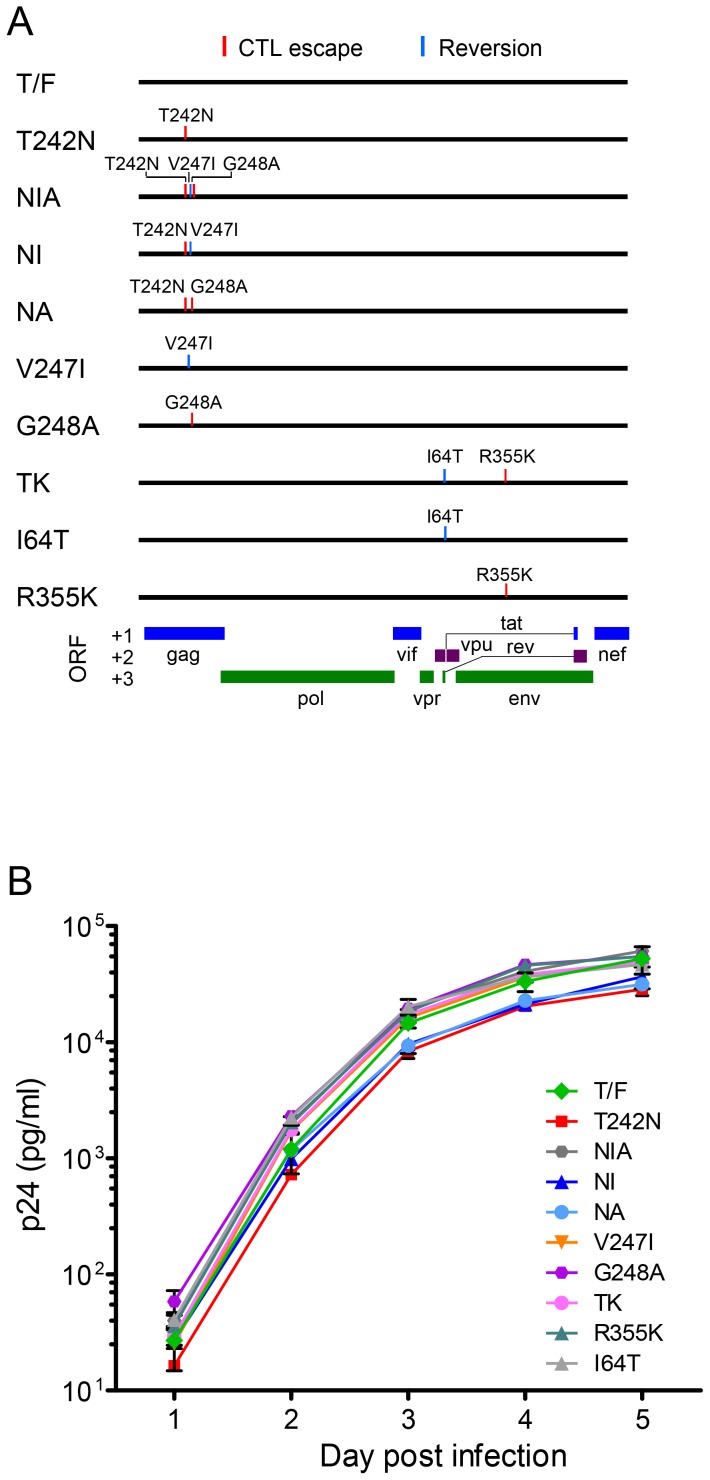
Generation of CH77 T/F mutants and their replication kinetics in CD4^+^ T cells. (A) Schematic presentation of mutations introduced into the CH77 T/F genome. (B) Replication kinetics of CH77 T/F viruses and its mutants. CD4^+^ T cells were infected with equal amount (1000 TCID_50_) of CH77 T/F virus or its mutants. Each virus was cultured independently in triplicates. The viral replication kinetics was determined by measuring the p24 concentration in the culture supernatant daily. Mean ± standard deviations are shown.

Our previous study showed that the NIA mutant, which contained three mutations (T242N, V247I and G248A) in the TW10 epitope in patient CH77 (Fig. 1A), was as fit as its cognate T/F virus, but the fitness of the T242N mutant, which differed from the T/F virus by only the T242N CD8^+^ T cell escape mutation, was severely impaired [25]. We also showed that an early mutant (TK), which contained a reversion mutation (I64T) in Tat/Rev and a CD8^+^ T cell escape mutation (R355K) in Env, was similarly fit to the CH77 T/F virus [25]. To understand whether these mutations, independently or together with other mutations, could affect the fitness of their cognate T/F virus or mutants selected by CD8^+^ T cell *in vivo*, we generated four single-mutation IMCs, each of which contained a single reversion mutation (V247I or I64T) or a single CD8^+^ T cell escape mutation (V247I, G248A or R355K) and two double-mutation IMCs, each of which contained two mutations (NI containing T242N and V247I, and NA containing T242N and G248A) (Fig. 2A). The replication kinetics of each virus was determined by measuring the p24 concentration daily in the supernatant of each independent virus culture (Fig. 2B). All viruses showed similar replication kinetics as we previously observed [25]. The viruses grew exponentially from day 1 to day 3, and then the replication rate slowed down at days 4 and 5 (Fig. 2B). The T242N, NI and NA mutants, which all contained the T242N CD8^+^ T cell escape mutation, replicated at slightly lower levels than other viruses between day 3 and day 5 (∼1.6-fold lower than the T/F virus).

**Figure 3:**
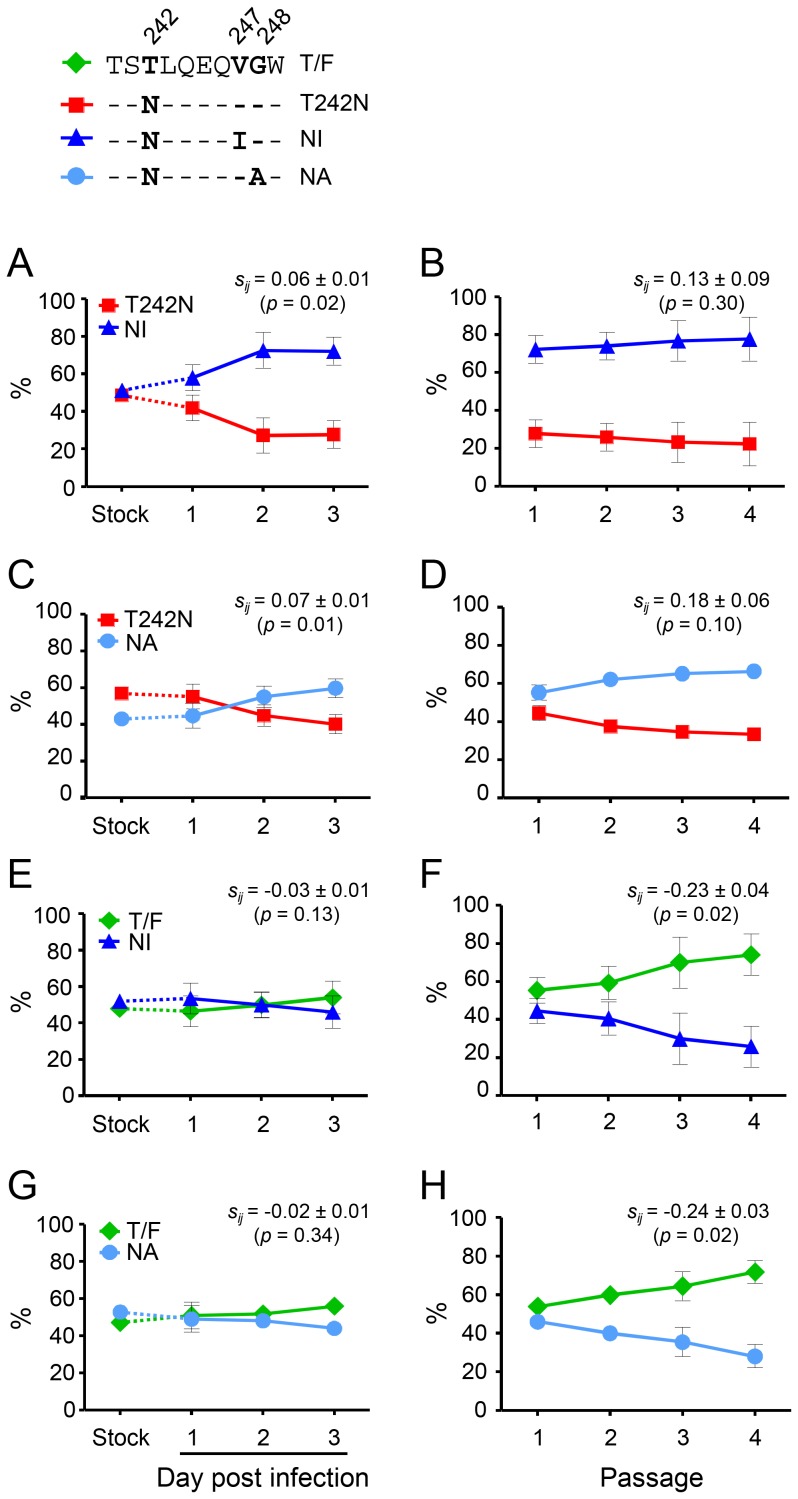
Partial restoration of the fitness loss of the T242N mutant by compensatory mutations. The relative fitness was determined between the T242N mutant and the NI (A and B) and NA (C and D) mutant as well as between the T/F virus and NI (E and F) or NA (G and H) mutant in the single-passage assay (left panels) and the multiple-passage assay (right panels). Same amount (5 ng p24) of each compared virus was mixed to infect 10^6^ of purified CD4^+^ T cells in triplicates. In the single-passage assay, the viruses were cultured for three days and the culture supernatant were harvested daily by completely replacing the medium. In the multiple-passage assay, 200 µl of cell-free virus harvested at day 3 at each passage was used to infect 10^6^ of fresh CD4^+^ T cells. The viruses were subsequently passaged three additional times. The percentage of each virus in the inoculum stock and the culture supernatant was determined by PASS. Mean ± standard deviations are shown. The relative fitness was determined by modeling the replication slope of each virus in the single- and multiple-passage assays as previously described [25].

To determine whether the V247I or G248A mutation alone could fully compensate the fitness loss due to the T242N mutation, we compared the NI or NA mutant to the T242N mutant (Fig. 3A–3D). In the single-passage assay, NI was 6±1% more fit than the T242N mutant (p = 0.02), while NA was 7±1% more fit than the T242N mutant (p = 0.01) (Fig. 3A and 3C). During three additional passages, both NI and NA mutants continuously outgrew the T242N mutant (Fig. 3B and 3D). In the multiple-passage assay, the relative fitness estimates have standard errors of about 0.08, and do not allow a quantitative estimate of fitness differences less than that.

We next investigated if the V247I or G248A mutation alone could fully restore the fitness of the T242N mutant to the T/F level. Both NI (s_ij_ = −0.03±0.01; p = 0.13) and NA (s_ij_ = −0.02±0.01; p = 0.34) were as fit as the T/F virus in the single-passage assay (Fig. 3E and 3G), similar to what we previously observed for the comparison between the T242N mutant and the T/F virus [25]. However, both NI and NA mutants were significantly less fit during four additional passages (Fig. 3F and 3H). The NI mutant was 23±4% less fit than the T/F virus (p = 0.02) and the NA mutant was 24±3% less fit than the T/F virus (p = 0.02). These results demonstrated that the V247I or G248A mutation alone could partially restore the fitness loss caused by the T242N mutation in the TW10 epitope, but only both together could fully compensate the fitness cost due to the T242N mutation as previously demonstrated [25].

### No measurable impact on the fitness of the cognate T/F virus was detected for the V247I or G248A mutation

**Figure 4:**
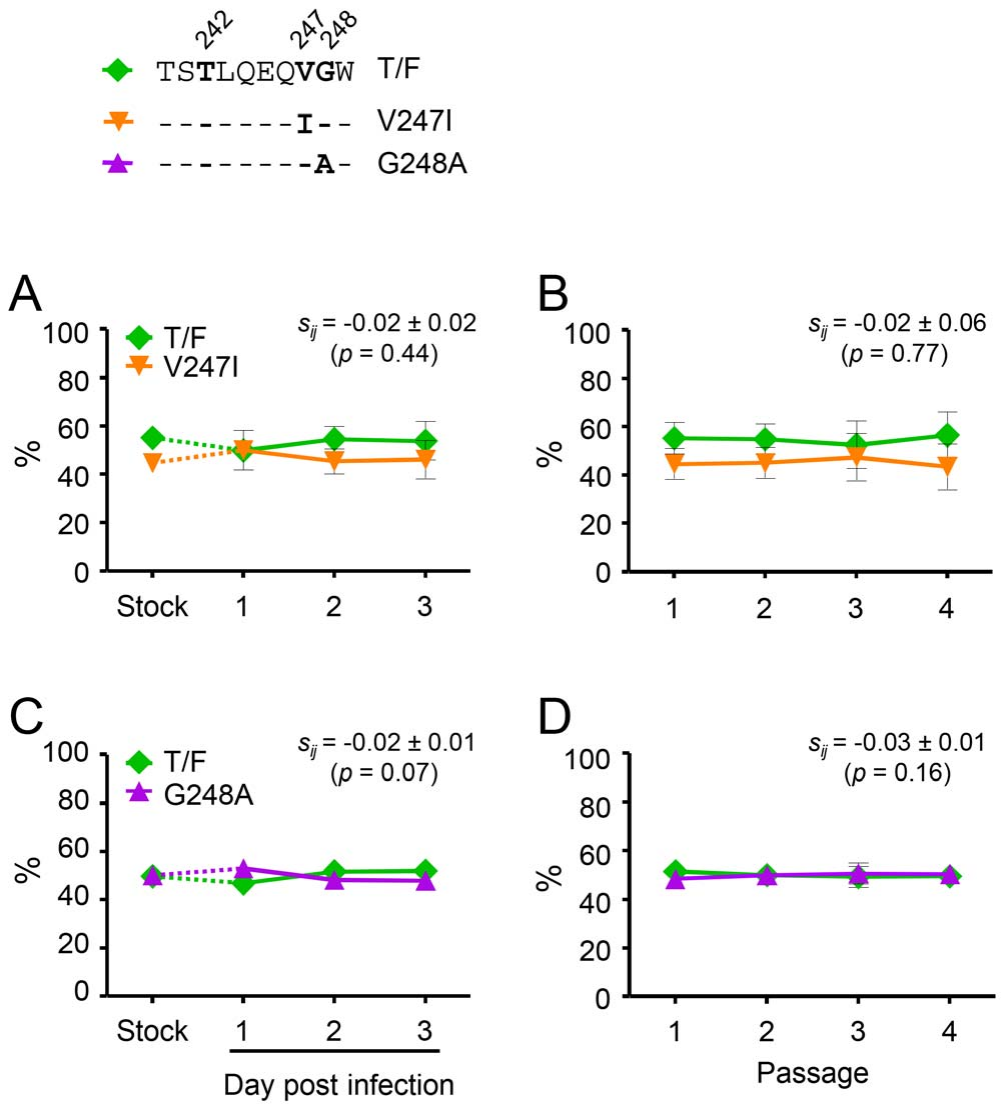
Impact of the V247I or G248A mutation alone on the fitness of their cognate T/F virus. The fitness impact of the V247I or G248A mutation alone was determined by comparing the mutant V247I (A and B) or G248A (C and D) to their cognate T/F virus in the single-passage assay (A and C) and the multiple-passage assay (B and D). The percentage of each virus in the inoculum stock and the culture supernatant was determined by PASS. Mean ± standard deviations are shown.

We next sought to determine whether the V247I or G248A mutation compensated for the fitness loss due to the T242N mutation because they could render their cognate T/F virus more fit. When the V247I mutant was compared with the T/F virus, no significant fitness differences were observed between the two viruses in both single-passage assay (*s_ij_* = −0.02±0.02; p = 0.44) and multiple-passage assay (*s_ij_* = −0.02±0.06, p = 0.77) (Fig. 4A and 4B). Thus, the V247I mutation, as a reversion mutation and partial CD8^+^ T cell escape mutation, had no measurable impact on the fitness of its cognate T/F virus.

The CD8^+^ T cell escape mutation G248A is commonly associated with T242N in subtype B virus; 44% (123 of 278) of the subtype B viral sequences with N242 also had A248 in the HIV Sequence Database (http://www.hiv.lanl.gov). Previous studies showed that the G248A mutation remained stable upon transmission to HLA-B57/5801 negative recipients, suggesting little or no fitness cost of this mutation *in vivo* [5]. Both fitness loss and slight fitness gain were observed for the G248A mutation when it was analyzed in a heterologous viral genome NL4-3 *in vitro* [16], [19], [39]. To more clearly define its impact on viral fitness, we determined the relative fitness between the G248A mutant and its cognate T/F virus. The result showed that the G248A mutant was as fit as the T/F virus in both single-passage (*s_ij_* = −0.02±0.01; p = 0.07) and multiple-passage assay (*s_ij_*_ = _0.03±0.01; p = 0.16) (Fig. 4C and 4D), indicating that the partial CD8^+^ T cell escape mutation G248A did not significantly affect the fitness of its cognate T/F virus.

### The reversion mutation I64T in Tat/Rev overlapping region had no measurable fitness cost

**Figure 5:**
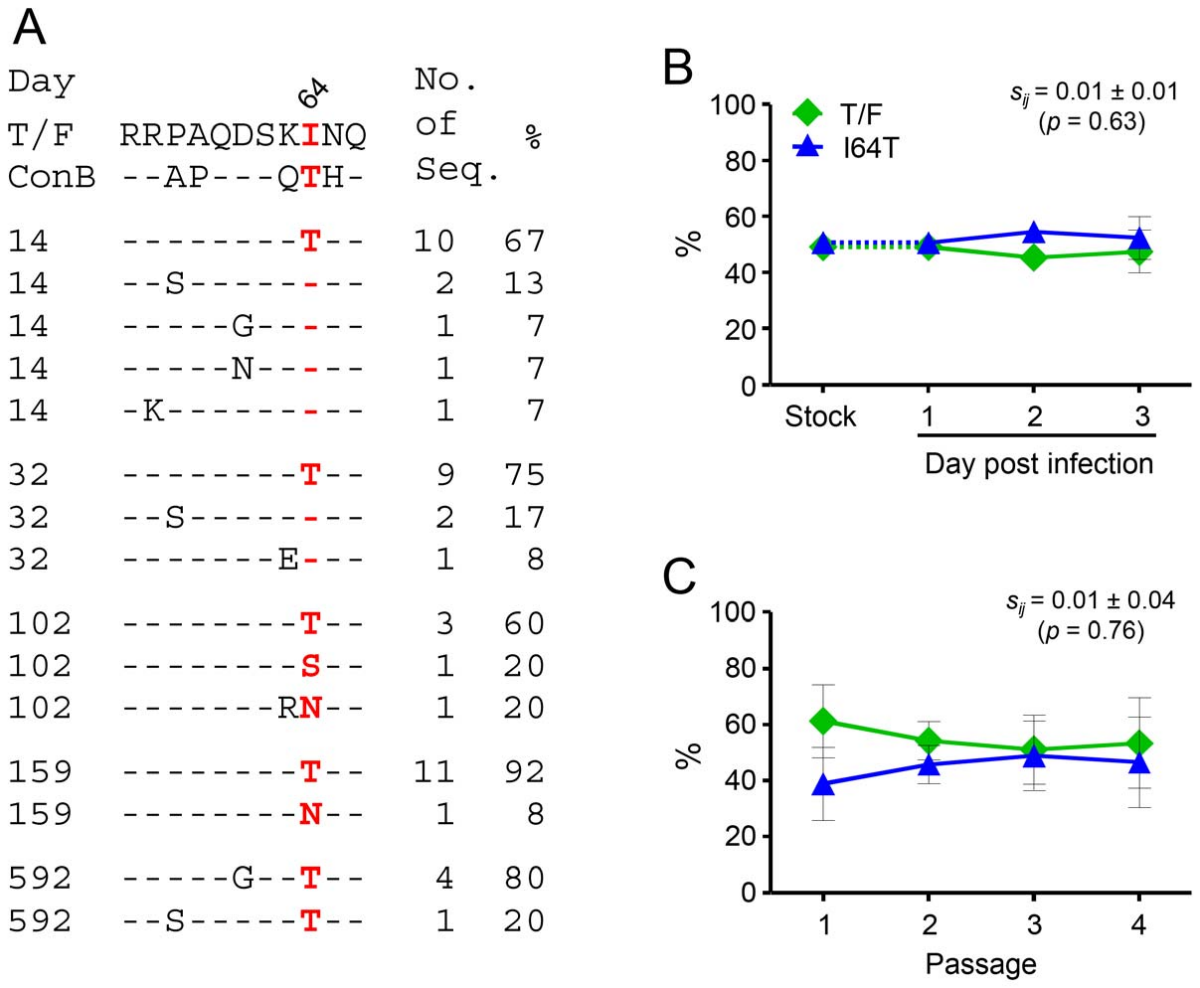
No fitness costs of the early reversion mutation I64T in Tat/Rev overlapping region. (A) Frequencies of the I64T mutation in the Tat protein at different time points (days post Fiebig stage I/II). The viral sequences obtained by SGA were compared to the T/F virus and the subtype B consensus sequence (ConB). Amino acid substitutions at the I64T mutation site were highlighted in red. Relative fitness of the I64T mutant was determined by comparing to the cognate T/F virus in the single-passage assay (B) and the multiple-passage assay (C). The percentage of each virus in the inoculum stock and the culture supernatant was determined by PASS. Mean ± standard deviations are shown.

The I64T reversion mutation in the Tat/Rev overlapping region was one of the two earliest mutations detected in CH77 [9]. It dominated the viral population (67%) as early as at day 14 post Fiebig stage I/II and remained predominant during the first two years of infection (Fig. 5A). No T cell responses were detected against the region containing this mutation in Tat in our previous study [9]. Although it was within a T cell epitope in the overlapping Rev coding region (Rev_9–26_), the mutation was synonymous in the *rev* gene [9]. Thus, the rapid reversion of Ile to Thr at position 64 in Tat could not be caused by the selection pressure from T cell responses. To test whether this reversion mutation had any impact on the viral fitness, we compared the viral fitness between the I64T mutant and the T/F virus. The I64T mutant was as fit as its cognate T/F virus in the single-passage assay (*s_ij_*_ = _0.01±0.01; p = 0.63) (Fig. 5B) and the multiple-passage assay (*s_ij_*_ = _0.01±0.04; p = 0.76) (Fig. 5C). This result showed that, in addition to the reversion mutation V247I, the reversion mutation I64T also did not cause measurable fitness costs in its cognate T/F virus in an *in vitro* fitness assay.

### The early partial CD8^+^ T cell escape mutation R355K in Env did not cause fitness loss

**Figure 6:**
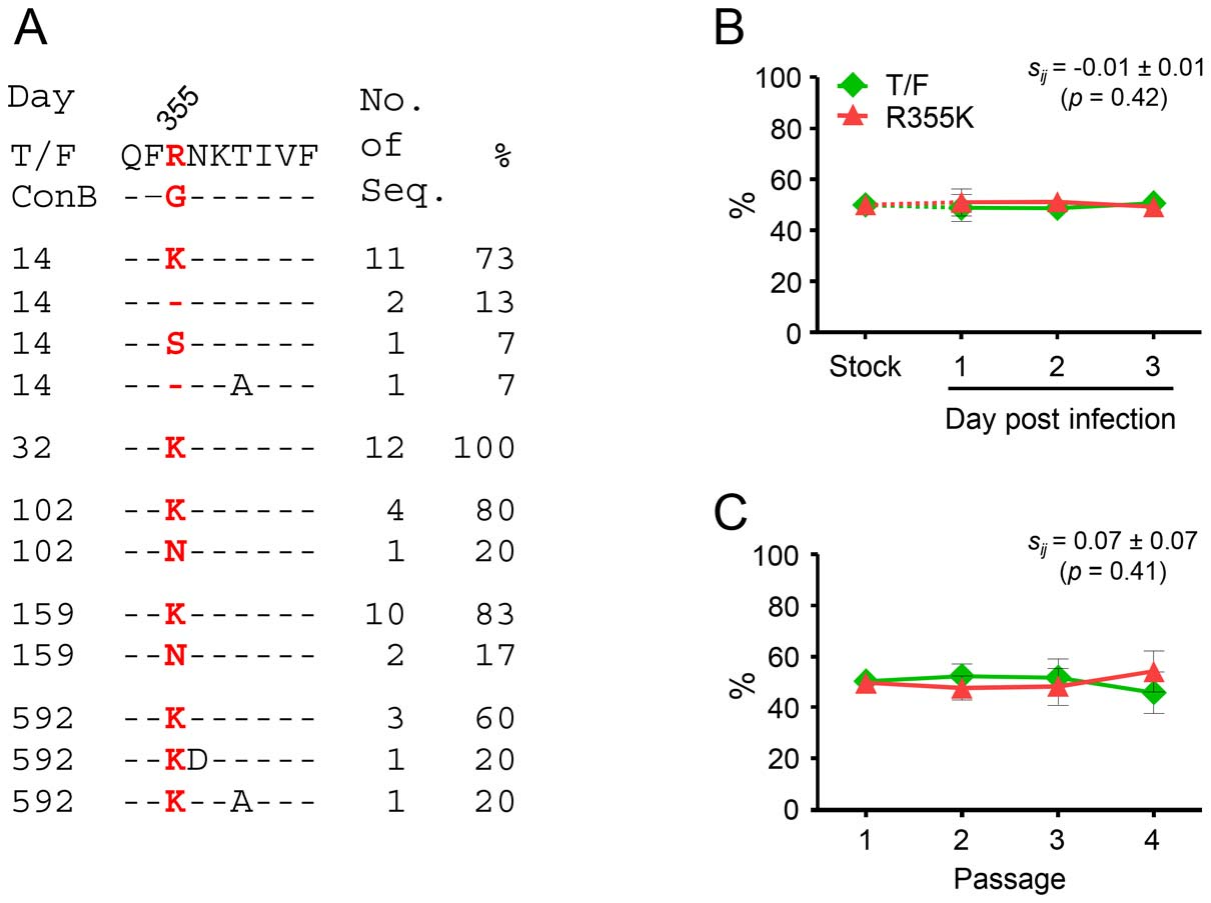
No fitness costs of the early CD8^+^ T cell escape mutation R355K in Env. (A) The frequency of the R355K mutation in the Env_352–360 _T cell epitope different time points (days post Fiebig stage I/II). The viral sequences obtained by SGA were compared to the T/F virus. Amino acid substitutions at the R355K mutation site were highlighted in red. Relative fitness of the R355K mutant was determined by comparing to the cognate T/F virus in the single-passage assay (B) and the multiple-passage assay (C) as described in Figure 3. The percentage of each virus in the inoculum stock and the culture supernatant was determined by PASS. Mean ± standard deviations are shown.

R355K was the earliest CD8^+^ T cell escape mutation in CH77 selected by T cell responses targeting the epitope Env_352–360_ [9]. It was selected as early as day 14 post Fiebig stage I/II, and then predominated the viral population at day 32 and all later time points (Fig. 6A). We previously showed that the mutant TK, which contained both R355K and I64T mutations, did not cause any fitness loss when compared to the T/F virus [25]. To further investigate whether this early CD8^+^ T cell escape mutation alone had any fitness cost, we compared the R355K mutant with the T/F virus. No significant impact on the viral fitness of the cognate T/F virus was detected in the single-passage assay (*s_ij_* = −0.01±0.01; p = 0.42) (Fig. 6B) and in the multiple-passage assay (*s_ij_*_ = _0.07±0.07; p = 0.41) (Fig. 6C).

### The mutations in the TW10 epitope did not cause significant structural changes

**Figure 7:**
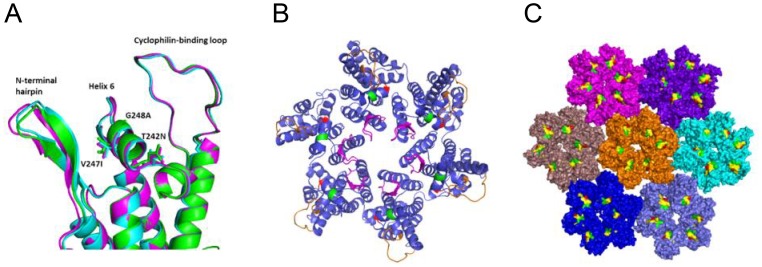
Structural modeling of mutations in the TW10 epitope in p24 Gag. (A) Homology models of the p24 monomer for the sequences of the T/F virus (cyan), the T242N mutant (magenta) and the NIA mutant (green) show similar structures of the helix 6 region with modest structural differences in the neighboring N-terminal hairpin and CypA binding loop. Side chains at mutation positions are shown as stick representation. (B) Mutations at position 242 (red) and positions 247/248 (green) in helix 6 were mapped to the hexameric p24 crystal structure [49]. The mutation positions did not occur at p24 subunit interfaces. Helix 6 is located between the N-terminal hairpin (magenta) and cyclophilin-binding loop (orange) on the surface of the hexamer. (C) Mutation at position 242 (red) and positions 247/248 (green) in helix 6 (yellow) were mapped to a hexamer of hexamers in the capsid assembly [40]. The T242N (red) and V247I/G248A (magenta) mutations face outward in the assembly and do not occur at hexamer-hexamer interfaces.

To understand whether the fitness cost and compensation of the T242N, V247I and G248A mutations could be explained by alterations in the structure, stability or assembly of the p24 protein, we modeled their potential impact on the p24 structure based on the recently published structural data [36], [40]. All three mutations were located in helix 6, which is situated between the N-terminal hairpin and the cyclophilin A (CypA)-binding loop on the surface of the N-terminal domain (NTD) on the exterior side of the capsid [36], [40] (Fig. 7A). Homology modeling of p24 monomers did not result in substantial structural differences in helix 6 between Thr in the T/F virus and Asn in the T242N mutant (0.45Å backbone atom RMSD), suggesting that the T242N escape mutation in the context of the full-length p24 is not expected to destabilize the helix 6 structure (Fig. 7A). In addition, only modest changes in the neighboring CypA-binding loop and N-terminal hairpin were observed in the T242N model (1.05 Å and 2.52 Å backbone atom RMSD, respectively). The modeling of all three mutations (T242N, V247I and G248A) also showed no significant perturbation of helix 6 (0.74 Å backbone atom RMSD), and only minor differences in the CypA loop and N-terminal hairpin compared to the T/F sequence model (0.89 Å and 1.06 Å backbone RMSD, respectively). Interestingly, the structural differences from the T/F model in the N-terminal hairpin region were smaller in the NIA model than in the T242N model.

Helix 6 is not located in the interface of p24 subunits in hexamers (Fig. 7B) or in inter-oligomer interfaces in a model of the capsid assembly [40] (Fig. 7C). Therefore helix 6 mutations were not expected to alter capsid stability by contributing unfavorable contacts across p24 interfaces. Taken together, the structural modeling results showed that the subtle structural changes caused by the mutations at positions 242, 247 and 248 may not affect the p24 stability or assembly.

## Discussion

We studied the impact of reversion and T cell escape mutations in their unmodified cognate T/F viral genome and found that reversion mutations did not cause measurable fitness gains in the cognate T/F virus but could compensate the fitness loss caused by the CD8^+^ T cell escape mutation T242N, and, unexpectedly, weak CD8^+^ T cell escape mutations could also partially compensate the fitness loss caused by the T242N mutation in the same epitope. Our findings demonstrate that the overall viral fitness is modulated by the complex interplay among compensatory, reversion and CD8^+^ T cell escape mutations to maintain the balance between immune escape and viral replication capacity.

Immune responses can exert strong selection pressure to select escape mutants that can quickly replace the sensitive viruses [4], [9], [13], [41]–[43]. Some selected mutations in the donors can revert back to the consensus sequence in the newly infected recipients [1]–[5], [9] and these reversion mutations have been considered to render the viruses more fit [1]–[9]. We studied two reversion mutations (V247I and I64T), but neither of them rendered the cognate T/F virus more fit as determined by the competitive PASS fitness assay. However, a small fitness gain of HIV-1 mutants can only significantly increase their proportion in the viral population after many infection cycles [44]. It took months for both the V247I and I64T reversion mutations to become fixed in the population from the first time of detection. Similar evolutionary dynamics were also observed for the reversion of the T242N mutation [5], [10]–[12]. These results suggest that the fitness advantage might be small for the reversion mutants.

The reversion mutation V247I, also a weak T cell escape mutation, could partially compensate the fitness loss due to the T242N mutation. The V247I mutation was selected by the early T cell responses. The T242N mutation that alone could cause significant fitness loss was selected by a late potent T cell response targeting the TW10 epitope [9]. Because the compensatory mutation V247I had been present in the majority of the viral population when the T242N was selected, the fitness loss caused by the T242N mutation would be readily alleviated by the preexisting V247I mutation. The G248A mutation also allowed the virus to partially escape from the early T cell responses. Conflicting results about fitness costs of the G248A mutation were reported in previous studies [16], [19], [39]. However, how it interacted with the T242N mutation and affected the viral fitness with or without the T242N mutation has not been studied. Our results unexpectedly demonstrated that both weak T cell escape mutations (V247I and G248A) could partially repair the fitness loss due to the CD8^+^ T cell escape mutation T242N. Since both the V247I and G248A mutations together could fully restore the significant fitness loss caused by the T242N mutation [25], the CD8^+^ T cell escape mutant containing both the fitness-impairing escape mutation (T242N) and compensatory mutations (V247I and G248A) could be as pathogenic as the wild type viruses. Thus, HIV-1 could use the compensatory and/or CD8^+^ T cell escape mutations that were preexisting and concurrently selected to maintain high replication capacity even when the CD8^+^ T cell escape mutations with significant fitness cost were selected. Both of the compensatory mutations V247I and G248A were first selected by T cell responses before the fitness-impairing T242N mutation in CH77. However, this might not necessarily indicate that the T242N mutation would only be selected after the V247I and/or G248A mutation selected, since the I247 was predominant (97.28%) while A248 was frequently detected (22.08%) among subtype B sequences in the Los Alamos HIV-1 sequence database showed. The finding that T cell escape mutations could act as compensatory mutations to restore fitness loss caused by another CD8^+^ T cell escape mutation demonstrated that HIV-1 developed a unique mechanism to maintain viral replication capacity by utilizing the immune selected mutations. These results indicated that the interplay among different mutations played an important role in maintaining viral fitness under the immune selection pressure during infection.

One CD8^+^ T cell escape mutation (R355K) in Env was selected by the T cell responses very early during infection. The lack of measurable fitness cost of the R355K mutation might facilitate its rapid escape kinetics *in vivo* and the quick predominance in the viral population throughout the follow-up period. This was in agreement with our *in vivo* observation that the R355K mutation persisted without reversion after the T cell responses targeting the Env_352–360_ epitope vanished [9]. The lack of measurable fitness cost for the R355K mutation was in consistent with previous observations that R355K escaped rapidly after transmission and was in a high entropy epitope [43], [45]. A previous study also showed that the CD8^+^ T cell escape mutations in Env, unlike most of those in Gag, did not have fitness costs [18]. The lack of detection of fitness costs of some T cell escape mutations suggested that the loss of T cell recognition did not always result in the fitness loss. However, we have found that neutralizing antibody escape mutations could result in various levels of fitness costs [41], [46]. Although the highly variable HIV-1 *env* gene may better tolerate immune escape mutations without loss of fitness, more T and B cell escape mutations need to be studied to fully understand the fitness costs of immune escape mutations in Env. It was also possible that both CD8^+^ T cell escape mutations (R355K and G248A) could cause fitness losses which were too small to be detected by the current fitness assays.

Structural mechanisms for reduction of viral fitness by helix 6 mutations have been hypothesized, including destabilization of the helix 6 and more distant effects on the p24 monomeric structure through conformational coupling of helix 6 to the N-terminal hairpin and CypA-binding loop [14], [15]. Our protein modeling analysis of helix 6 showed that only subtle structural differences were observed between the T/F and all three mutations (T242N, V247I and G258A) in structural models of p24. Moreover, helix 6 was not located in the interface of p24 subunits in hexamers or in inter-oligomer interfaces in a model of the capsid assembly [40]. Therefore, the mutations in helix 6 were not expected to alter capsid stability by contributing unfavorable contacts across p24 interfaces. It was unclear whether these small differences in the N-terminal hairpin were merely reflective of the modeling capturing an inherent flexibility in the N-terminal hairpin or if they suggested a potential structural mechanism for escape and compensation whereby p24 oligmerization could be affected by subtle changes in N-terminal hairpin positioning. However, the fitness cost and compensation of fitness loss of those three mutations might not be solely explained by those subtle predicted structure alterations. It is likely that the fitness cost of the T242N mutation and the compensatory effect of the V247I and G248A mutations were mediated by altering its interaction with CypA or other host factors important for virus replication, instead of dramatically disturbing the capsid stability or assembly. The increased dependency on CypA of the T242N mutant and compensation of the fitness loss of the T242N mutation by mutations in the CypA-binding loop [15], [47], as well as the potential effect on the packing of the CypA binding loop by amino acids at position 248 [14] support this hypothesis.

Our results have several important implications. First, the fitness cost of a CD8^+^ T cell escape mutation will depend on the concurrent compensatory mutations and the context of the viral genome. This suggests that the potential clinical benefits of the CD8^+^ T cell escape mutations should be analyzed together with compensatory mutations. The recently described quantitative fitness landscape method may be helpful to better understand the impact of mutational couplings in the context of entire protein sequence [48]. Second, a full restoration of viral fitness by compensatory mutations suggests that the CD8^+^ T cell escape mutants can be as pathogenic and transmissible as the wild type viruses. Third, reversion mutations may only render the virus slight more fit so they eventually outgrow T cell escape mutant viruses over time. Such subtle fitness gain may be too small be detected by the current *in vitro* fitness assays. Finally, identification of T cell epitopes that confer significant fitness costs which cannot be restored by compensatory mutations may assist in design of more effective T cell-based HIV-1 vaccines.
